# Controlling inversion disorder in a stoichiometric spinel magnet

**DOI:** 10.1073/pnas.2208748119

**Published:** 2022-10-18

**Authors:** Margarita G. Dronova, Feng Ye, Scott E. Cooper, Anjana Krishnadas, Christina M. Hoffmann, Yuita Fujisawa, Yoshinori Okada, Daniel I. Khomskii, Yejun Feng

**Affiliations:** ^a^Okinawa Institute of Science and Technology Graduate University, Onna, Okinawa 904-0495, Japan;; ^b^Neutron Scattering Division, Oak Ridge National Laboratory, Oak Ridge, TN 37831;; ^c^II. Physikalisches Institut, Universität zu Köln, D-50937 Köln, Germany

**Keywords:** single-crystal growth, antiferromagnetic spinel, inversion disorder, neutron magnetic diffuse scattering, antiferroelectricity

## Abstract

In the study of frustrated quantum magnets, it is essential to be able to control the nature and degree of site disorder during the growth process, as many measurement techniques are incapable of distinguishing between site disorder and frustration-induced spin disorder. Pyrochlore-structured spinel oxides can serve as model systems of geometrically frustrated three-dimensional quantum magnets; however, the nature of the magnetism in one well-studied spinel, ZnFe_2_O_4_, remains unclear. Here, we demonstrate simultaneous control of both stoichiometry and inversion disorder in the growth of ZnFe_2_O_4_ single crystals, directly yielding a revised understanding of both the collective spin behavior and lattice symmetry. Crystals grown in the stoichiometric limit with minimal site inversion disorder contravene all the previously suggested exotic spin phases in ZnFe_2_O_4_. Furthermore, the structure is confirmed on the $F\bar{4}3m$ space group with broken inversion symmetry that induces antiferroelectricity. The effective tuning of magnetic behavior by site disorder in the presence of robust antiferroelectricity makes ZnFe_2_O_4_ of special interest to multiferroic devices.

## Significance

While disorder is an inalienable characteristic of real crystalline materials, the capability of controlling various types of disorder often strongly influences our understanding of science and the advancement of technology. Magnetic spinel represents a class of materials with a pyrochlore-structured sublattice to potentially host three-dimensional spin frustration but is strongly influenced by the inversion disorder of two similarly sized cation species. While it remains challenging to experimentally differentiate these two characteristics, here we mitigate the disorder issue at the crystal growth stage. Our independent control of both stoichiometry and inversion disorder clarifies both magnetism and structure in a spinel oxide of interest for seven decades.

To control disorder is of foundational importance in the advancement of both science and technology, best exemplified by the continued improvement of the size and quality of silicon single crystals as an enabling capability for the current information age (1). In frustrated and quantum magnetism (2–5), crystalline disorders, such as admixed site occupancies, vacancies, and impurities, present a significant challenge to experimental studies of exotic spin phases. While disorder can sometimes enable exotic physics, for instance a modest level of disorder can in theory promote spin entanglement (6) and disorder played a major role in revealing the quantum Hall effect (7), more typically real materials exhibit excessive levels of disorder that would suppress quantum effects (4, 8). While disorder and frustration lead to different global spin states (2), it remains experimentally challenging to differentiate a quantum entangled spin state from an ensemble of disordered spin singlets (5). Disorder is most effectively mitigated during the growth of single crystals, exemplified by the continuing improvement of GaAs heterogenous junctions leading to advances in the study of the fractional quantum Hall effect (9).

**Fig. 1.**
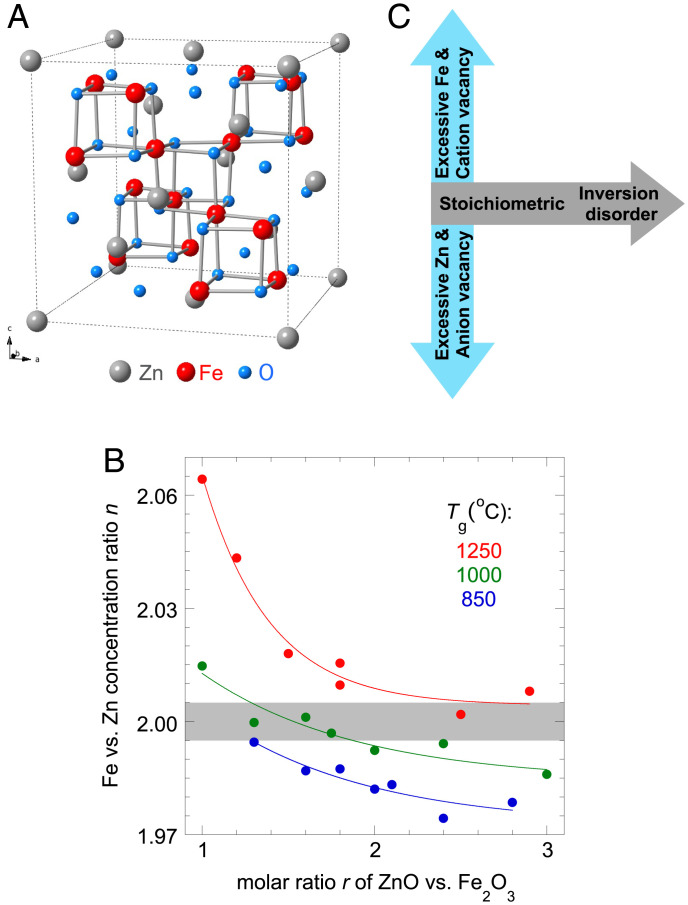
Controlling disorder in spinel ZnFe_2_O_4_. (*A*) Schematic of the normal spinel unit cell in the $Fd\bar{3}m$ space group, highlighting the clusters of Fe (red) tetrahedra and their O (blue) neighbors. (*B*) XRF-WDS measured Fe vs. Zn concentration ratio *n* of all our grown crystals (*Materials and Methods*), as a function of the two growth parameters *T*_g_ and *r*. The gray region (1.995 ≤n≤ 2.005) represents the stoichiometric limit in our characterization. (*C*) A schematic of the two-dimensional phase space of disorder in a normal spinel. The horizontal axis represents the stoichiometric condition, with varying degrees of inversion disorder between *A* and *B* sites. The orthogonal direction represents excessive Fe or Zn concentrations away from the stoichiometric condition and vacancy disorders of either cations or anions (assumed to be mutually exclusive). Inversion disorder also exists in the off-stoichiometric condition but is not well defined. This phase diagram, as a reinterpretation of *B*, summarizes the consistent understanding of all our characterizations in the current study.

The pyrochlore lattice structure is a model host of potential geometric spin frustration in three dimensions (2, 3). With *s* = 1/2 spins, the lattice can potentially host many different types of quantum spin liquids that preserve both the lattice symmetry and time-reversal symmetry (10). Among the three crystalline classes that contain a pyrochlore sublattice, spinel, Laves, and pyrochlore (3), the spinel *AB*_2_O_4_ of normal condition (Fig. 1) is unique in that with edge-sharing *B*O_6_ octahedra, the *B-*O*-B* angle is near 90°, leading to both direct hopping exchange and multiple superexchange scenarios between *B* ions with a hierarchy of interaction strengths of both ferromagnetic and antiferromagnetic types (11, 12). As competing interactions strongly depend on both the elemental species and the *B-*O*-B* angle (11, 12), there potentially exist many scenarios of strong spin frustration and liquid-like orbital and magnetic ground states in pyrochlore spinels. However, cation imbalance, oxygen deficiency, and inversion disorder are prevalent practical concerns in pyrochlore oxides (3, 8). As the *A* and *B* ions in the spinel structure are of similar sizes, within ∼±15% of each other (13), spinel oxides are prone to site disorder. The normal spinel (*A*^2+^)[*B*^3+^]_2_O_4_ has *A* ions fully occupying 8*a* (tetrahedral) sites of the $Fd\bar{3}m$ space group (Fig. 1*A*), and the inverse spinel (*B*^3+^)[*A*^2+^*B*^3+^]O_4_ has *A* ions randomly occupying half of the 16*d* [octahedral] sites. The inversion disorder, defined as the parameter *λ* of (*A*_1−_*_λ_B_λ_*)[*A_λ_B*_2−_*_λ_*]O_4_ with (tetrahedral) and [octahedral] sites (14), varies continuously in the range 0≤λ≤1 between these two limiting cases. We note that the inversion disorder is only well defined when stoichiometry is strictly kept; i.e., the *B* ion concentration is twice that of the *A* ion. This inversion disorder between similarly sized *A* and *B* ions (13, 15) makes spinels a challenging class of geometric spin frustration materials to grow and characterize (14).

ZnFe_2_O_4_ exemplifies the challenge of high-quality crystal growth in spinel oxides. Despite being studied since the 1950s (16–27), literature reports of the magnetic properties of ZnFe_2_O_4_ are not consistent. While many ^57^Fe Mossbauer measurements (23, 25) confirm that the iron in ZnFe_2_O_4_ is entirely Fe^3+^ ions, these can be introduced into various types of disorder by thermal and/or mechanical preparation processes such as heating, annealing, powdering, and ball milling (21, 23, 25, 26). Neutron magnetic diffraction (16, 19, 20, 22–25, 27) often demonstrated a superposition of a broad diffusive profile and a peak around the (1/2, 1, 0) wave vector at ∼4 K, indicating varying degrees of short- and long-range spin orders in the bulk (19, 23), with the long-range order attributed to antiferromagnetism below *T*_N_ ∼ 10 K (18, 19). Magnetic susceptibility measurements often found a positive (ferromagnetic) Curie–Weiss temperature *T*_CW_ (23–25), despite the assumption that the system is antiferromagnetic, giving rise to an unclarified implication of spin frustration (24, 27). Alternating current magnetic susceptibility studies have shown that the cusp temperature can be frequency dependent (25), suggesting the existence of very slow spin dynamics, similar to a spin glass (27) or a spin liquid (24). However, the peak in the magnetic susceptibility in the direct current (DC) limit (24, 25, 27) is not consistent in temperature with the peak of the magnetic heat capacity, which is consistently observed below 10 K as either a broad bump or a relatively sharp divergence (17, 21). We note that most measurements in the literature, such as in refs. 16–19, 21–23, and 25–27, were carried out on powder specimens of ZnFe_2_O_4_ rather than single crystals.

Here, we demonstrate growth strategies that control both the stoichiometry and the levels of inverse disorder in single-crystal ZnFe_2_O_4_ and utilize this control to resolve these decades-long ambiguities. We show that stoichiometry in spinel ZnFe_2_O_4_ can be maintained over a narrow trajectory across the growth parameter space, leading to crystals of different levels of inversion disorder. In the disorder-free limit, stoichiometric ZnFe_2_O_4_ single crystals demonstrate a consistent divergence at *T*_N_ = 9.9 K in both magnetic susceptibility and heat capacity and exhibit long-range antiferromagnetic order with no trace of magnetic diffuse scattering. These results show that the exotic spin phases suggested in the literature should instead be attributed to disorder effects. The experimentally observed antiferromagnetic Curie–Weiss temperature *T*_CW_ is now consistent with the theoretically expected nearest-neighbor interaction between Fe ions on octahedral sites. Further, ZnFe_2_O_4_ can be categorized as type I multiferroic material with antiferroelectricity in the lattice space group $F\bar{4}3m,$ regardless of the level of inversion disorder. Our study serves as a benchmark for the level of quality control attainable in magnetic spinel oxides and promotes pyrochlore-structured oxides as a fertile research field of frustrated and quantum magnetism and strongly correlated electrons.

## Results

### Disorder Control at the Growth Stage.

*AB*_2_O_4_ spinel oxides can be grown by many different techniques over a wide temperature range, such as floating zone (28), high-temperature solution (24), Czochralski, and chemical vapor transport (29) for single crystals; solid-state reaction (25) for polycrystals and powders; and hydrothermal and solution–gelatin (21, 26) for nanoparticles. Here we focus on high-temperature solution growth (*Materials and Methods*), as this technique can be carried out over a large range of temperatures with many choices of solvents (30) and can produce single crystals at 1 mm size or larger, a critical threshold for many characterization techniques and applications. Growing crystals in ambient atmosphere also could protect the Fe^3+^ ions from reducing to the Fe^2+^ state.

One major concern of high-temperature solution growth is the potential inclusion of flux and/or its ionic components in the form of impurities. We have grown ZnFe_2_O_4_ single crystals with three different types of solvents: anhydrous Na_2_B_4_O_7_, triple-oxide BaO/Bi_2_O_3_/B_2_O_3_ mixture (30), and a binary mixture of Li_2_B_4_O_7_ and Na_2_B_4_O_7_ at their eutectic point (31). We found that among these, Na_2_B_4_O_7_ was the best choice based on evaluations of the crystals’ magnetic properties. For Na_2_B_4_O_7_, both Na^+^ and B^3+^ ions are significantly different in size from Zn^2+^ and Fe^3+^ ions, and thus they are not included as cation impurities without causing structure instability (13, 15). The results discussed below focus on single crystals grown in Na_2_B_4_O_7_ solvent. As Na_2_B_4_O_7_ is typically regarded as viscous, we rotated the crucibles inside the furnaces at a constant angular speed (*Materials and Methods*). The rotation greatly enhances the solution homogeneity and significantly reduces the number of nucleation centers on the crucible’s inner surface, leading to larger and more consistent crystals.

Unlike most growth procedures that first utilize a solid-state reaction to produce spinel-structured powder before dissolving that powder in the solvent to grow single crystals (24), we instead directly dissolve ZnO and Fe_2_O_3_ powders in molten Na_2_B_4_O_7_ at the highest temperature of growth *T*_g_ to form ZnFe_2_O_4_, following their reported binary phase diagram (32). The disorder in the resulting spinel single crystals is thus controlled by two growth parameters, the highest temperature *T*_g_ experienced and the molar ratio *r* = (mole of ZnO)/(mole of Fe_2_O_3_) in the initial powder mixture (*Materials and Methods*). Single-phase ZnFe_2_O_4_ growth has been observed over most of the parameter space of *T*_g_ from 1,250 °C to 850 °C and *r* from 1.0 to 3.0. However, for *T*_g_ = 850 °C and *r* < 1.30, an additional single-crystal phase of Fe_2_O_3_ emerges; no growth of ZnO single crystal has been observed in this range. Without a loss of generality, we limit our discussion to remain within the parameter space of single-phase spinel ZnFe_2_O_4_.

### Bulk Elemental Analysis and the Map of Disorders.

The large range in the (*T*_g_, *r*) parameter space we explored for the growth control is expected to introduce multiple types of disorder (14), such as nonstoichiometry between the Fe and Zn cations, cation or anion vacancies, and admixed cation site occupancies (inversion). Given the potential changes of physical properties when single crystals are turned into powder form (21, 23, 25, 26), all our characterizations are performed on single crystals. We first examine the overall Fe vs. Zn concentration ratio *n* in bulk crystals, using elemental analysis based on wavelength-dispersive X-ray fluorescence spectroscopy (XRF-WDS) (*Materials and Methods*). Using hard X-rays instead of electrons to excite XRF, our measurements are sensitive to elements at a depth of 20 to 40 μm in the bulk, limited only by the absorption of Zn and Fe *K*α X-ray fluorescence in the material.

The Fe vs. Zn concentration ratio *n* of our grown crystals is systematically examined and plotted against *T*_g_ and *r* in Fig. 1*B*. For growths with identical *T*_g_, *n*(*r*) evolves monotonically with *r* as expected, reflecting the controlling condition of the growths. This evolution allows a finite range of *r* for each *T*_g_ to bring *n* to the stoichiometric limit, defined here as being within ±0.25%  of 2.000 (gray shaded region in Fig. 1*B*). For *T*_g_ = 1,250 °C, all crystals have excess iron and only a very large *r* ≥ 2.5 can bring *n* to ∼2.005. At *T*_g_ = 1,000 °C, an increasing *r* drives the crystals from Fe excess to Zn excess, reaching the stoichiometric condition for *r* between 1.6 and 1.8. At *T*_g_ = 850 °C, *r* = 1.3 is the best condition to grow crystals at the stoichiometric limit of *n* ∼ 1.995, and larger *r* results in crystals with excess Zn. These optimal growth conditions of stoichiometric crystals are strongly correlated with the magnetic characterizations discussed below.

In nonstoichiometric crystals with *n* deviating from 2, excessive Zn introduces an oxygen deficiency, whereas excess Fe causes cation vacancies on both the *A* and *B* sites. As our crystals are grown in molten oxides at ambient air pressure, we assume anion and cation vacancies do not happen simultaneously but instead are mutually exclusive. For stoichiometric crystals, we expect from entropy considerations that there should be varying levels of inversion disorder associated with the different growth temperatures *T*_g_; we expect that crystals grown with *r* = 1.3 at *T*_g_ = 850 °C would have the least amount of inversion disorder. The two growth parameters *r* and *T*_g_ that guided the plot of *n* in Fig. 1*B* are thus mapped onto a two-dimensional phase space of sample quality, denoted by two orthogonal axes of cation/anion vacancies (with the stoichiometric condition at the origin) and inversion disorder from zero to high (Fig. 1*C*).

### Disorder Effects in Magnetic Susceptibility.

**Fig. 2.**
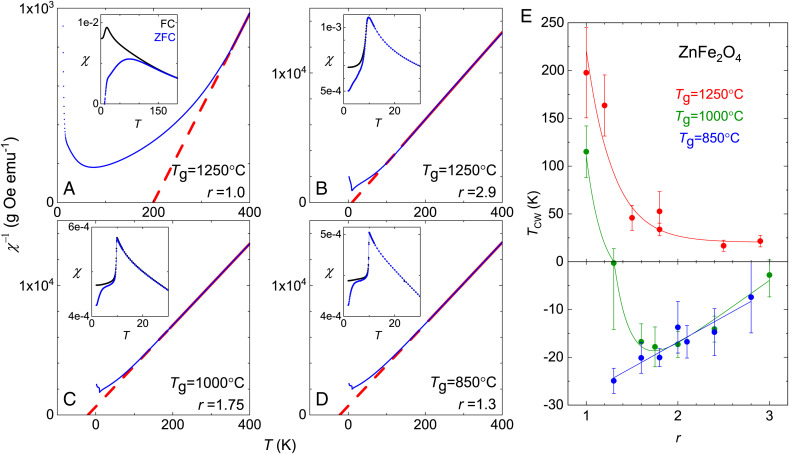
Disorder influence on magnetic susceptibility and Curie–Weiss temperature. (*A*–*D*) Inverse magnetic susceptibility 1/χ (blue points) of ZnFe_2_O_4_ single crystals grown with various *T*_g_ and ZnO vs. Fe_2_O_3_ molar ratio *r*. The high-temperature region of 1/χ is fitted to the Curie–Weiss law (red solid and dashed lines) for the extraction of both *T*_CW_ and the local moment size. (*Insets*) Zoomed-in views of the magnetic susceptibility χ around *T*_N_ for both FC and ZFC conditions (*Materials and Methods*). *A*–*D* correspond to crystals listed in *SI Appendix*, Fig. S1 *A*–*D*, respectively. (*E*) Evolution of *T*_CW_(*r*) for three different *T*_g_. Each data point represents the average of 10 to 40 individual crystals including those in *A*–*D* (*Materials and Methods*); error bars represent the 1σ SD of the mean. The solid lines are guides to the eye. The negative segment of the *y* axis is expanded to highlight *T*_CW_’s approach to the clean limit.

The charge-based elemental analysis discussed above does not provide insight into the role of disorder on the magnetism of ZnFe_2_O_4_. Instead, we use the magnetic characteristics as an indicator of the site disorder of the Fe ions. In Fig. 2, we first examine the evolution of the DC magnetic susceptibility χ with *T*_g_ and *r*. For the most disordered single crystals, with *T*_g_ = 1,250 °C and *r* = 1.0, the zero-field–cooled χ(*T*) has a large spectral weight that peaks around 80 K (Fig. 2 *A*, *Inset*), similar to that of nanocrystals and strained powder (23, 25, 26). The 1/χ(*T*) does not show linear Curie–Weiss (CW) behavior for any temperature range below 400 K. Instead, a tangential fit of 1/χ(*T*) at the highest temperature suggests a ferromagnetic CW temperature *T*_CW_ ∼ 200 K (Fig. 2*A*). As *r* is increased, *T*_CW_ monotonically decreases (Fig. 2), correlated with the decrease of the Fe vs. Zn concentration ratio *n* toward stoichiometry (Fig. 1). For all our crystals grown with *T*_g_ = 1,250 °C, *T*_CW_ remains positive (Fig. 2*E*). When *T*_g_ is reduced to 1,000 °C, *T*_CW_(*r*) no longer has a monotonic trend but instead reaches a minimum at *r* = 1.6 to 1.8 with an antiferromagnetic *T*_CW_ ∼ −20 K (Fig. 2*E*), identical to the *r* values where the Fe vs. Zn concentration ratio *n* becomes stoichiometric (Fig. 1). For crystals grown with *T*_g_ = 850 °C, the lowest *T*_CW_ ∼ −25 K is achieved at *r* = 1.3, again coinciding with stoichiometric results from the elemental analysis (Fig. 1). For grown crystals of the same *T*_g_, *T*_CW_(*r*) is always the lowest when they are stoichiometric.

For crystals deviating from the stoichiometric condition, the increased *T*_CW_(*r*) suggests that excessive Fe or Zn concentrations (Fig. 1), with concurrently increased vacancies, increase the level of spin disorder, obscuring the traditional interpretation of a Curie–Weiss analysis by violation of its mean-field assumption. On the other hand, 1/χ(*T*) of stoichiometric crystals grown at three different *T*_g_ differ in a subtler manner (Fig. 2 *B*–*D*). All three 1/χ(*T*) are similar at high temperature (∼400 K). Apart from the change of the Curie–Weiss temperature from 25 to −25 K (Fig. 2*E*), the largest difference is seen in the peak region of χ(*T*) around *T*_N_; the critical divergence in χ(*T*) sharpens, while the amplitude gradually reduces in value (Fig. 2 *B*–*D*, *Insets*). The Curie–Weiss temperatures and slopes of stoichiometric crystals grown at 1,000 °C and 850 °C are essentially identical. The linear slopes of Curie–Weiss fits of these two crystals (Fig. 2 *C* and *D*) yield a consistent local spin moment of 5.53(1) μ_B_ per Fe^3+^ ion, within 7% of the theoretical value 5.92 μ_B_ for an *s* = 5/2 state of 3*d*^5^ Fe^3+^. Our results suggest that most of the inconsistencies in the χ(*T*) behavior reported in the literature (21, 23–27) are likely due to off-stoichiometry concentrations of the samples.

### Spin Correlation Revealed by Neutron Magnetic Diffuse Scattering.

The spin correlations and disorder at microscopic length scales can be directly visualized by neutron magnetic diffuse scattering (*Materials and Methods*), allowing us to directly search for signatures of inversion disorder in stoichiometric crystals. Here, we compare two stoichiometric single crystals (*SI Appendix*, Figs. S1–S3), grown at *T*_g_ = 1,250 °C and 1,000 °C, whose overall magnetism is characterized by positive (∼25 K) and negative (∼ −22 K) *T*_CW_, respectively.

**Fig. 3.**
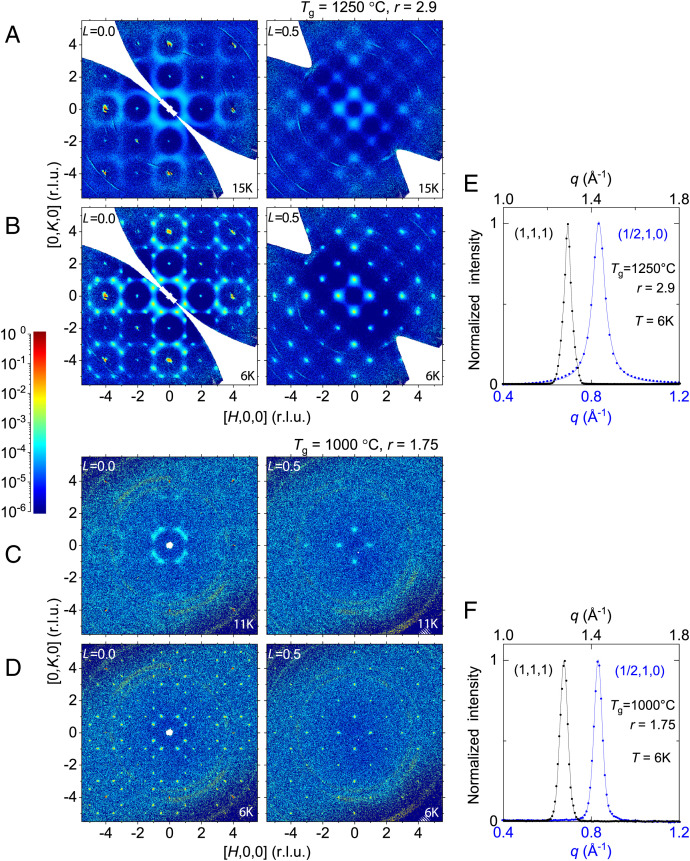
Contrast of spin correlations between disordered and ordered stoichiometric crystals. Unsymmetrized scattering intensities, measured from two sets of crystals grown from (*A* and *B*) *T*_g_ = 1,250 °C (crystal assembly in *SI Appendix*, Fig. S1*E*) and (*C* and *D*) *T*_g_ = 1,000 °C (single crystal in *SI Appendix*, Fig. S1*C*), respectively (*Materials and Methods*), are presented as *H*–*K* plane slices at *L* = 0.0 and 0.5. All intensities have been integrated over a thickness of 0.05 r.l.u. along *L*. For each sample, the magnetic scattering is contrasted between temperatures (*A* and *C*) above and (*B* and *D*) below the antiferromagnetic transition. As the samples have different masses and mosaicity, the intensities (color bar) in *A*–*D* are normalized by equalizing the reciprocal-space integrated intensities of lattice reflection families <1, 1, 1> and <2, 2, 0> at 6 K (*Materials and Methods*). The plotted magnetic-scattering intensities are further isolated by subtracting the background and lattice diffraction pattern at high temperature, 100 K for (*A* and *B*) and 25 K for (*C* and *D*), where the magnetic diffuse scattering is nonexistent (*Materials and Methods* and *SI Appendix*, Figs. S2 and S3). Circular rings in all panels are due to powder diffraction by the aluminum holders. (*E* and *F*) Radial (longitudinal) scans across both the lattice reflection (1, 1, 1) and the magnetic reflection (1/2, 1, 0) for the same two samples (*Materials and Methods*). The solid line of the lattice shape in *E* is a guide to the eye, as its form is not described by the pseudo-Voigt function because of the convolution of lattice constant distribution of 30 individual crystals and the instrument resolution. The magnetic reflection in *E* is fitted with Lorentzian. Both curves in *F* are pseudo-Voigt with 80 and 65% of weight in Gaussian for the lattice and the magnetic reflections, respectively. Both curves of the lattice and the curve of magnetic reflection in *F* have identical FWHM as they are dominated by the instrument resolution.

For stoichiometric crystals grown at *T*_g_ = 1,250 °C (Fig. 3 *A* and *B*), a large amount of magnetic diffuse scattering is seen both below and above *T*_N_. Across *T*_N_, only a fraction of the magnetic diffuse scattering at 15 K (Fig. 3*A*) sharpens to form long-range ordering at 6 K, and a large amount of spectral weight remains diffusive (Fig. 3*B*). The diffuse scattering pattern of our disordered, stoichiometric crystals is very similar to that reported in ref. 24. By contrast, magnetic scattering from our stoichiometric crystal grown at *T*_g_ = 1,000 °C is totally different. The magnetic diffuse scattering at 11 K (Fig. 3*C*), only about 1 K above *T*_N_, is much weaker than the magnetic diffuse scattering of the more disordered stoichiometric crystal at 15 K (Fig. 3*A*). Below *T*_N_, all diffusive spectral weight disappears, and only sharp, resolution-limited (Fig. 3*F*), magnetic reflections are observed at 6 K (Fig. 3*D*). This indicates that the magnetic diffuse scattering in Fig. 3*C* originates from true critical fluctuations, whereas those in Fig. 3 *A* and *B* are mostly due to spin scattering from a high level of static inversion disorder.

From the stoichiometric *T*_g_ = 1,000 °C crystal in the disorder-free limit (Fig. 3*D*), we confirm that the primary wave vector of the magnetic order is (1/2, 1, 0). While the spin structure will be refined in detail in a future publication, spin correlation lengths in these crystals are readily extracted from a radial scan across the (1/2, 1, 0) reflection in comparison with lattice correlation lengths measured at the (1, 1, 1) reflection (Fig. 3 *E* and *F*). For the stoichiometric crystal grown at *T*_g_ = 1,250 °C (Fig. 3 *A* and *B*), the longitudinal line shape is of Lorentzian form, and the full width at half maximum indicates that spin correlations exponentially decay over a spatial length scale of 30 Å, or four unit cells. On the other hand, in the stoichiometric crystal grown at *T*_g_ = 1,000 °C (Fig. 3 *C* and *D*), both lattice and magnetic longitudinal line shapes are identical and mostly Gaussian, indicating the line width is dominated by the instrument resolution and the spin correlation length is beyond 48 Å. This comparison of correlation lengths suggests that the stoichiometric crystal grown at 1,250 °C has at least four times as many pinning centers, and correspondingly higher inversion disorder, per unit volume compared to the stoichiometric crystal grown at 1,000 °C. With the unnoticeable amount of magnetic diffuse scattering observed in Fig. 3 *D* and *F*, neutron magnetic diffuse scattering is unlikely to further differentiate the two stoichiometric crystals approaching the disorder-free limit, grown at *T*_g_ = 1,000 and 850 °C, respectively.

Neutron diffuse scattering also provides an opportunity to evaluate the potential existence of spatial correlations between disordered sites. In *SI Appendix*, Fig. S4, we plot *H*–*K* plane patterns at *L* = 12, 13, and 14 reciprocal lattice unit (r.l.u.) for the set of stoichiometric crystals grown at *T*_g_ = 1,250 °C (*SI Appendix*, Fig. S1*E*), which has the largest amount of inversion disorder among three specimens. At this range of large transferred momentum, magnetic diffuse scattering is expected to vanish. The lack of any diffusive pattern across the Brillouin zone indicates the inversion disorder in our crystals is uncorrelated in space.

### Magnetic Heat Capacity in the Clean Limit.

**Fig. 4.**
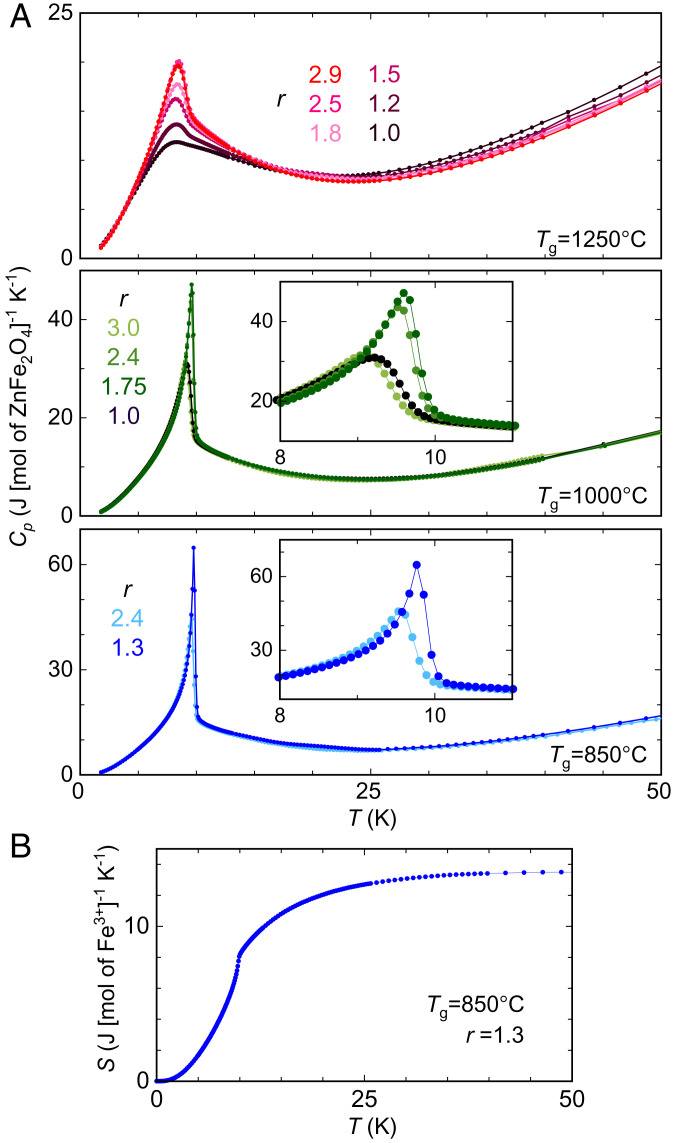
Influence of both off-stoichiometry and inversion disorder on heat capacity. (*A*) Heat capacity *C*_p_(*T*) of ZnFe_2_O_4_ single crystals as a function of the growth parameters *T*_g_ and *r*. (*Insets*) Zoomed-in views of *C*_p_(*T*) around *T*_N_. The sharpest *C*_p_ curve in each panel is from stoichiometric crystals. The sharpness of the most divergent *C*_p_ increases with lowering *T*_g_, due to the reduction in inversion disorder. (*B*) Temperature evolution of the magnetic entropy per mole of Fe^3+^ moments, integrated after removing phonon contribution from the total heat capacity (*Materials and Methods*).

Heat capacity *C*_p_(*T*) provides an independent and highly sensitive evaluation of inversion disorder in stoichiometric crystals approaching the disorder-free limit (Fig. 4). For crystals grown with the same *T*_g_, the sharpest *C*_p_(*T*) curves (Fig. 4*A*) always belong to those at the stoichiometric condition (Fig. 1) and the lowest *T*_CW_ in magnetic susceptibility (Fig. 2). Conversely, the broadened *C*_p_(*T*) in off-stoichiometric crystals presumably is due to the magnetic order being pinned at shortened correlation lengths by an increasing number of vacancies, preventing spins from having a sharply defined phase transition.

For stoichiometric crystals grown with reduced *T*_g_, *C*_p_(*T*) progressively diverges. While magnetic susceptibility shows only a minimal difference between stoichiometric crystals grown with *T*_g_ = 1,000 and 850 °C (Fig. 2 *C* and *D*), divergences of *C*_p_(*T*) at *T*_N_ are distinctly different (Fig. 4*A*). Since excessive Zn cations are necessary in the starting solution to bring the spinel crystals to stoichiometry, we assume the excessive-to-normal Zn ion ratio (*r* − 1) is proportional to the level of inversion disorder, as both should reduce to zero in the ideal situation. We thus estimate that the level of inversion disorder differs by a factor of 2 to 3 between these two sets of stoichiometric crystals with *r* of 1.75 and 1.3, respectively. For the crystals grown with *T*_g_ = 850 °C and *r* = 1.3, *C*_p_(*T*) reaches a peak value of 65.1 J⋅mol^−1^⋅K^−1^ or equivalently 15.6 Cal⋅(mole of ZnFe_2_O_4_)^−1^ (Fig. 4*A*), much more singular than the best *C*_p_(*T*) of 9.2 Cal⋅mol^−1^⋅K^−1^ reported in the literature (17). Here, for stoichiometric single crystals grown with *T*_g_ = 850 °C, both *C*_p_(*T*) and χ(*T*) diverge consistently at *T*_N_ (Figs. 2*D* and 4*A*). The magnetic heat capacity, integrated after subtraction of the phonon background, reveals a magnetic entropy of 13.5 J⋅mol^−1^⋅K^−1^, which is slightly smaller than *R*ln6 = 14.9 J (mole of Fe^3+^)^−1^ K^−1^ (Fig. 4*B*), consistent with a classical spin *s* = 5/2 in this spinel.

### Lattice Symmetry of the Stoichiometric Crystals.

**Fig. 5.**
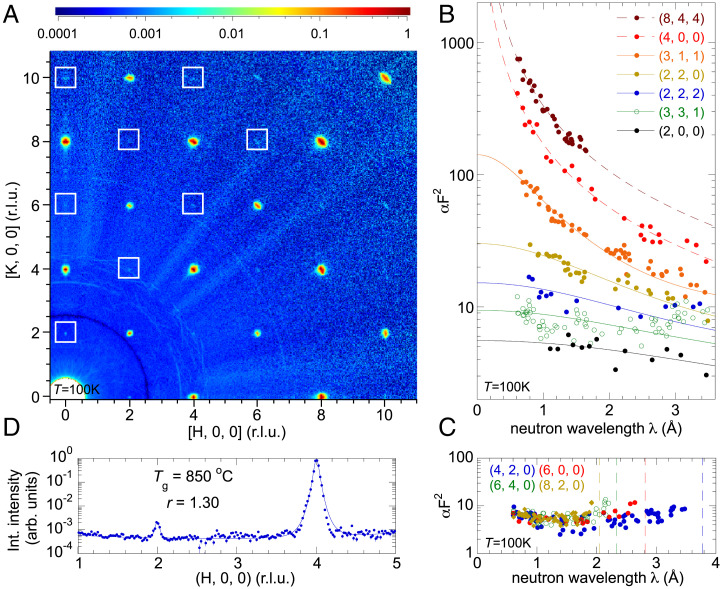
Lattice space group revealed by continuous-wavelength neutron diffraction. (*A*) A slice of the *H–K* plane scattering intensity at the *L* = 0 position for the single crystal grown in the (*T*_g_ = 850 °C, *r* = 1.3) condition (*SI Appendix*, Fig. S1*D*). The graphic is generated from symmetrized neutron-scattering data in the reciprocal space, and several white boxes highlight the forbidden reflections of $Fd\bar{3}m,$ the traditionally understood space group of ZnFe_2_O_4_. Instead, these observed reflections satisfy the selection rules of the space group $F\bar{4}3m.$ (*B* and *C*) Wavelength dependence of scattering intensities $\alpha F_{\mathrm{hkl}}^{2}(\lambda )$ of several unique reflection families. In *B*, intensities of several reflection families are fitted for the extinction effect (solid lines) (*Materials and Methods*) to extract the extinction-free value $\alpha F_{\mathrm{hkl}}^{2}(0)$ at the zero-wavelength limit. For several reflection families of very high intensity, the extinction formula does not apply. Instead, a power-law form of $\alpha F_{\mathrm{hkl}}^{2}\left(\lambda \right)∼\lambda^{-\beta }$ (dashed lines) fits the data well with an exponent β∼1.5. *C* emphasizes the wavelength dependence of the forbidden reflections of the space group $Fd\bar{3}m.$ The vertical dashed lines indicate the long-wavelength diffraction limits of each family. The absence of diffraction events beyond these wavelength limits indicates that multiple scattering does not contribute to these reflections. (*D*) A radial (longitudinal) scan across both the (2, 0, 0) and (4, 0, 0) reflections, generated from *A*. Lines of both reflections have comparably sharp profiles, indicating a long-range lattice order.

**Fig. 6.**
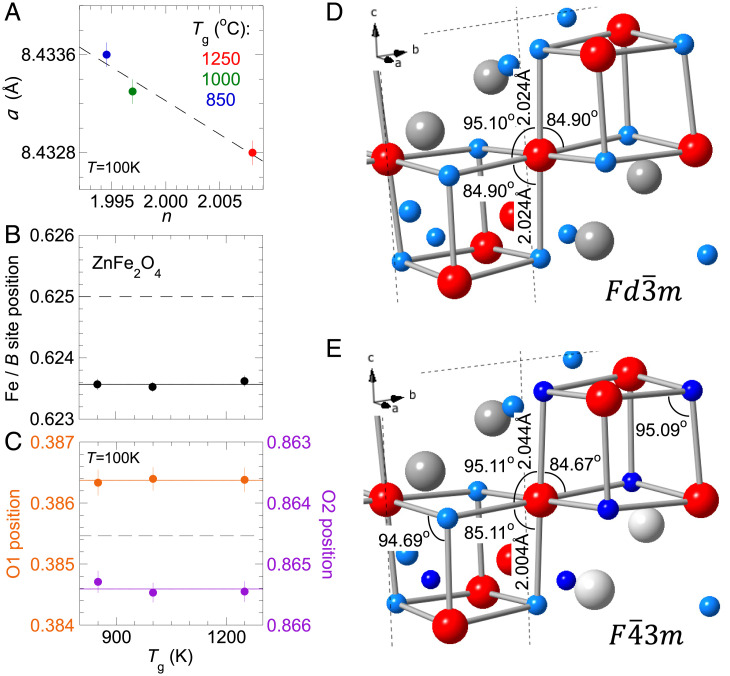
Refined lattice parameters. (*A*) The cubic lattice constants of three stoichiometric crystals with slight difference in stoichiometry are plotted against the deviation of Fe vs. Zn concentration *n* from a perfect 2.000. The cubic lattice constants were determined during the establishment of UB orientation matrix (*Materials and Methods*). (*B* and *C*) Atomic position parameters for (*B*) *B* site (or Fe) and (*C*) two oxygens at 16e positions in $F\bar{4}3m$ space group are plotted for three stoichiometric crystals against the growth temperature *T*_g_. All three crystals demonstrate consistent values and significant deviations from the expected values (two dashed lines) of the $Fd\bar{3}m$ space group. *C* is constructed such that if ZnFe_2_O_4_ is in the $Fd\bar{3}m$ space group, both oxygen positions would overlap on the graph (summing to 1.25). Complete refinement results are listed in *SI Appendix*, Table S1. (*D* and *E*) Comparison of Fe-O bond lengths and angles of the FeO_6_ cage, situated between the adjacent Fe tetrahedra, using either (*D*) $Fd\bar{3}m$ or (*E*) $F\bar{4}3m$ space groups, respectively. The color assignment of atoms is the same as in Fig. 1. For $F\bar{4}3m$ space group, two different shades of blue and gray are used to indicate the two sites of O and Zn, respectively.

Given the importance of lattice symmetry in the exploration of quantum spin liquids (10), the three sets of stoichiometric ZnFe_2_O_4_ crystals, grown under different (*T*_g_, *r*) conditions, also provide a unique opportunity to examine the sensitivity of lattice characteristics to the level of inversion disorder. The lattice structure is characterized at the time-of-flight white-beam neutron diffractometer TOPAZ (33, 34) (*Materials and Methods* and Figs. 5 and 6), which records the timing of each scattered neutron at the detector so that the wavelength is well determined. This feature allows diffraction measurements with a continuous spectrum of neutron wavelengths, providing unique advantages in resolving some key issues as discussed below.

For all three sets of crystals, the reconstructed diffraction patterns in the three-dimensional reciprocal space confirm the cubic symmetry (*Materials and Methods*) and clearly demonstrate no reflection at mixed even/odd indexes, indicative of a face-centered–cubic Bravais lattice (Fig. 5*A* and *SI Appendix*, Fig. S4). The classical understanding of spinel ZnFe_2_O_4_ is of space group $Fd\bar{3}m$ (#227) (20, 22, 24). However, there is a clear set of nonvanishing (*h*, 0, 0) reflections with *h* = 4*m* + 2 and (*h*, *k*, 0) reflections with *h* + *k* = 4*m* + 2 (*m* being an integer) observed in all three stoichiometric crystals with different levels of inversion disorder (Fig. 5 and *SI Appendix*, Figs. S2–S4). These reflections are forbidden in space group $Fd\bar{3}m$ but are allowed in its maximal nonisomorphic subgroup $F\bar{4}3m$ (#216). Within the face-centered–cubic structure, the presence of (*h*, 0, 0) reflections rules out two other maximal nonisomorphic subgroups $F4_{1}32$ (#210) and $Fd\bar{3}$ (#203) (35). As all scattering events at TOPAZ have clearly resolved wavelengths, the observation cannot be attributed to harmonics, i.e., neutrons of half- or quarter-wavelengths that diffract at higher orders (36). Mixed-index reflections such as (1, 1, 0) or (3, 1, 0), indicative of potential issues with harmonics, were not observed in our data (Fig. 5*A*). These “forbidden” reflections such as (2, 0, 0) and (4, 2, 0) were observed over a wide range of incident neutron wavelengths with effectively constant intensities (Fig. 5 *B* and *C* and *Materials and Methods*), inconsistent with an origin of multiple scattering. Furthermore, they are observed only for wavelengths *λ* < 2*d*, where *d* is the *d*-spacing of the specific reflection. A multiple-scattering process combining two lower wavevectors, such as (6, 4, 0) = (3, 3, 1) + (3, 1, −1), would allow the diffraction event to be observed with neutrons of wavelength *λ* > 2*d*. Here, all scattering events are consistent with a single diffraction process (Fig. 5 *B* and *C*). The linewidth of a radial scan through the (2, 0, 0) reflection is indicative of a long-range order of the lattice, as it is as narrow as a radial scan of the main (4, 0, 0) reflection (Fig. 5*D*). Furthermore, all diffraction patterns at TOPAZ are consistent with the reconstructed reciprocal lattice patterns for magnetic diffuse-scattering study at CORELLI (Fig. 3 and *SI Appendix*, Figs. S2–S4), where the elastic nature of scattering is differently constrained through a cross-correlation mechanism (37). The (2, 0, 0) reflection was also observed in neutron single-crystal scattering using a triple-axis spectrometer in ref. 24 but neglected. ZnFe_2_O_4_ thus is attributed to space group $F\bar{4}3m.$ Among subgroups of $Fd\bar{3}m$ that also possess the face-centered–cubic Bravais lattice, *F23* (#196) is the only other space group that would conform to the observed diffraction pattern. However, *F23* is a subgroup of $F\bar{4}3m,$ and they differ only at 48*h* sites, while atoms in an *AB*_2_O_4_ spinel crystal occupy 4*a*, 4*c*, and 16*e* sites in the setting of $F\bar{4}3m.$ So there is no need to refine to a space group of lower symmetry than $F\bar{4}3m.$

For our crystals measured with neutron wavelengths from 0.6 to 3.5 Å (*Materials and Methods*), the diffraction intensities demonstrate a strong extinction effect even from a crystal as small as 0.7 mm in diameter (Fig. 5*B* and *SI Appendix*, Fig. S1*D*). While some reflections of medium and low intensity can be fitted to the standard formula of extinction (38) (*Materials and Methods*), strong reflections with an extinction suppression beyond a factor of 10 cannot be properly corrected (Fig. 5*B*). Overall, only ∼75% of the reflection families are used for refinement after applying the extinction correction (*Materials and Methods*). Modeling the extinction in Fig. 5*B* allows for a highly precise extraction of the structure factors $F_{\mathrm{hkl}}^{2}(\lambda =0).$

We first refine ZnFe_2_O_4_ in the $Fd\bar{3}m$ space group; the sole oxygen position parameter *u* = 0.38546 ± 0.00011 is consistent with the values reported in refs. 20 and 22. On the other hand, refining a spinel in the $F\bar{4}3m$ space group involves three parameters (*SI Appendix*, Table S1), one for the *B* site position that controls the breathing distortion of the pyrochlore sublattice and two additional oxygen position parameters along the (1, 1, 1) direction that are derived from the oxygen position parameter *u* of the $Fd\bar{3}m$ space group. In Fig. 6, all lattice parameters of the $F\bar{4}3m$ space group are plotted for all three stoichiometric crystals, labeled by the growth temperature *T*_g_. While the parameters are consistent across all three crystals, they clearly deviate from those of the $Fd\bar{3}m$ space group, especially the *B*-site position (Fig. 6*B*). In all three stoichiometric ZnFe_2_O_4_ crystals, the breathing distortion of the pyrochlore lattice remains stable and is not sensitive to the level of inversion disorder. The Fe-O bond lengths between neighboring Fe tetrahedra differ by 2.0% in the $F\bar{4}3m$ space group (Fig. 6*E*).

## Discussion

Our analysis indicates that a strong inversion disorder is responsible for both the ferromagnetic *T*_CW_ and strong spin diffuse scattering of static nature in many existing studies of ZnFe_2_O_4_ (16, 19, 20, 22–25, 27). In the presence of inversion disorder, the exchange interaction between Fe^3+^ ions on *B* and *A* sites is likely ferromagnetic and strong. On the other hand, the exchange interaction between *B* sites is predominantly through Fe-O-Fe bonds at near 90° angle (Fig. 6*E*). For Fe^3+^ ions, the *s* = 5/2 spin state indicates that the five 3*d* electrons are spread among both *t*_2g_ and *e*_g_ levels; there exist several different types of exchange processes involving both types of orbitals (11, 12). Similar to the analysis of edge-sharing FeO_6_ octahedra in pyroxenes (11), the direct Fe^3+^-Fe^3+^ hopping exchange interaction of the *t*_2g_-*t*_2g_ ($d_{xy}-d_{xy}$) channel within the plane of Fe-shared O-O edge-Fe should be antiferromagnetic but relatively weak, as 3*d* orbitals become small in Fe and the overlap is reduced (in comparison to light 3*d* elements such as Ti). For superexchange interactions, both the *t*_2g_-*t*_2g_ ($d_{yz}-p_{z}-d_{xz}$) and *t*_2g_-*e*_g_ ($d_{xy}-p_{x}-d_{2x^{2}-y^{2}-z^{2}}$) channels should be antiferromagnetic. For the *e*_g_-*e*_g_ ($d_{2y^{2}-x^{2}-z^{2}}-p_{y}/p_{x}-d_{2x^{2}-y^{2}-z^{2}}$) channel, the exchange interaction is ferromagnetic but very weak as the ∼95° Fe-O-Fe angle in ZnFe_2_O_4_ (Fig. 6*E*) is close to the boundary value of 97° for an ferromagnetic-to-antiferromagnetic (FM-AF) cross-over (11). Another ferromagnetic exchange channel involving two *p* states of oxygen is of *t*_2g_-*t*_2g_ ($d_{xy}-p_{x}/p_{z}-d_{xz}$) type but it is again very weak. Overall, there are many exchange pathways of the antiferromagnetic type and only a few of the ferromagnetic type, and the antiferromagnetic type *t*_2g_-*e*_g_ is expected to be the strongest among all. So, the total exchange interaction between Fe^3+^ spins should be antiferromagnetic. With the antiferromagnetic *T*_CW_ observed in our crystals (Fig. 2*E*), the experiment and theory are now qualitatively consistent.

Most spinel oxides are of the $Fd\bar{3}m$ space group, and the $F\bar{4}3m$ space group deduced here would break the inversion symmetry in creating Fe tetrahedra of alternating sizes (Fig. 6). Several spinel oxides, such as MgAl_2_O_4_ and MgV_2_O_4_, have been placed in the $F\bar{4}3m$ space group by direct diffraction techniques (39, 40). The crystal structure of ZnFe_2_O_4_ has been debated in the literature. While many references (20, 22) categorize it in the $Fd\bar{3}m$ space group, it has been suggested to belong the $F\bar{4}3m$ space group based on indirect evidence (41, 42). Crystals of proper stoichiometry and a minimal level of vacancies and inversion disorder are recognized as a key prerequisite to address this space group issue (43). Here, our systematic characterization of disorder as a function of growth conditions and diffraction studies using stoichiometric crystals in the disorder-free limit allows us to directly determine the space group assignment (Fig. 6). The alternatively sized tetrahedra in the $F\bar{4}3m$ space group can be visualized as the *B*-site ions of the $Fd\bar{3}m$ space group shifting toward and away from the centers of neighboring tetrahedra, forming an all-in–all-out type of displacement and associated local electric dipole moments. ZnFe_2_O_4_ thus exhibits antiferroelectricity from a breathing pyrochlore lattice in the $F\bar{4}3m$ space group (43), an electric dipole equivalent of the all-in–all-out antiferromagnetic state on the pyrochlore lattice. Here the antiferroelectricity in ZnFe_2_O_4_ is rather insensitive to the level of inversion disorder (Fig. 6), while the magnetic properties can be effectively switched between purely antiferromagnetic in disorder-free crystals and ferromagnetic or spin-glass type in highly site-disordered specimens (23, 25, 26). Although antiferromagnetism and antiferroelectricity exist independently in ZnFe_2_O_4_ as type I multiferroicity, the unique nature of site-disorder–controlled magnetic characteristics in the robust presence of antiferroelectricity makes ZnFe_2_O_4_ of potential application interest to multiferroic devices.

Previous experiments on Cr^3+^-based spinels (44) and analytical theory (45) have suggested Heisenberg spins interacting through antiferromagnetic exchange on a breathing pyrochlore lattice can form exotic ground states such as a classical spin liquid. As the breathing distortion of ZnFe_2_O_4_ lattice creates only a 2% variation of Fe-Fe distances in the small and large tetrahedra, we expect the nearest-neighbor Fe-Fe exchange interactions remain antiferromagnetic. The long-range magnetic order in ZnFe_2_O_4_ could be influenced by the next–nearest-neighbor interaction that is known to be important in spinels but was not considered by the theory (45). Both LiGaCr_4_O_8_ and LiInCr_4_O_8_ spinels of breathing pyrochlore lattices in ref. 44 exhibit inconsistent peak temperatures between the magnetic heat capacity and the magnetic susceptibility. While this inconsistency was attributed to the opening of a spin gap at temperatures higher than that of the long-range spin order (44), our current work demonstrates that such a discrepancy might not be intrinsic and can be removed through the reduction of disorder in single crystals.

Minimizing the inversion disorder in stoichiometric crystals is an important concern in the search for true spin frustration in quantum magnets. Here, our growth strategy allows a wide range of control over both the imbalance of initial nutrients composition and the adjustable temperature range, leading to a full exploration of the parameter space for crystals in the asymptotic disorder-free limit. Spinel oxides represent an important class of materials hosting three-dimensional geometrical spin frustrations. Exploration of this class of materials in the disorder-free limit, in the presence of intertwined spin, orbital, charge, and lattice degrees of freedom, is poised to lead to a cornucopia of discoveries in both strongly correlated electrons and quantum magnetism.

## Materials and Methods

### Single-Crystal Growth.

ZnFe_2_O_4_ spinel single crystals were synthesized using the high-temperature solution growth method. Powders of reagents ZnO (Sigma Aldrich; 99.99%) and Fe_2_O_3_ (Sigma Aldrich; 99.995%) and solvent anhydrous Na_2_B_4_O_7_ (Sigma Aldrich; >99%) were ground together. The initial powder mixture was loaded in a 20-mL platinum crucible, tightly covered by a platinum lid, heated to *T*_g_ at a rate of 50 °C/h, and held at *T*_g_ for 12 h. The ratios of solvent to solute, defined as *s* = (mol Na_2_B_4_O_7_)/(mol 3*d* cations Zn and Fe), were maximized from solubility studies to achieve solute saturation at each *T*_g_; *s* =0.94 for *T*_g_ = 1,000 °C, and *s* =1.4 for *T*_g_ = 850 °C. The crucibles were rotated inside the box furnaces throughout the synthesis process, with a rate of 96 rpm for *T*_g_ = 850 °C and 48 rpm for *T*_g_ = 1,000 °C. For *T*_g_ = 1,250 °C, either no rotation or a rotation rate of 24 rpm was used. ZnFe_2_O_4_ single crystals grow during the slow-cooling process, with a rate of −2 °C/h in the temperature range of 1,250 to 950 °C for *T*_g_ = 1,250 °C, −1.2 °C/h in the range of 1,000 to 800 °C for *T*_g_ = 1,000 °C, and −0.26 °C/h in the range of 850 to 750 °C for *T*_g_ = 850 °C, respectively. Afterward, the furnace was cooled down to room temperature naturally. Octahedron-shaped crystals (*SI Appendix*, Fig. S1) were extracted by etching in 7% HCl solution at 150 °C.

### Elemental Analysis.

The concentration ratio *n* of Fe and Zn in grown single crystals was determined by XRF-WDS (ZSX Primus II; Rigaku Corp.), using characteristic Fe-*K*α and Zn*-K*α fluorescent lines. The fluorescence was excited by X-rays generated from a Rh target. A 25-μm-thick aluminum filter was inserted in the incident beam path to block the direct heat from the X-ray tube to protect the crystals from overheating. To achieve reliable statistics of the Fe/Zn fluorescence intensity ratio, crystals from the same synthesis batch were densely packed but not overlapped in a disk form of 8 mm diameter, attached by a thin layer of silicone grease to a glass plate free of iron and zinc content. The top crystal surfaces were kept clean, flat, and parallel to the horizontal plane. To reduce spurious signals and increase spatial averaging, the sample holder was rotated with a speed of 30 rpm. The calibration of the Fe/Zn fluorescence ratio was carried out by measuring pressed pellets of mixed Fe_2_O_3_ and ZnO powders of seven different stoichiometric ratios from 1.96 to 2.08, in a disk form of 1 mm thick and 8 mm diameter, under the same measurement condition as the grown crystals.

### Magnetic Susceptibility.

The DC magnetic susceptibility χ was measured in a commercial magnetic property measurement system (MPMS3; Quantum Design Inc.), based on the superconducting quantum interference device (SQUID) technique. Individual single crystals were cooled to the base temperature of 1.8 K either in zero field (ZFC) or under a magnetic field of 100 Oe (FC); all were measured during the warming process under a 100-Oe field. Before the measurement, the remanent field in the MPMS magnet was cleansed to about 1 Oe by field oscillation. *T*_CW_ was calculated by the standard method of a linear fit to the Curie–Weiss law over the high-temperature portion of the inverse susceptibility 1/χ. The temperature range of the fitting was determined by gradually expanding the lower boundary of the fitting range from 400 K down, to the extent that the quality of the linear fit could be either improved or maintained. The temperature range of fitting is typically above 5|*T*_CW_| to justify the application of a Curie–Weiss analysis, except for two batches of the most disordered crystals grown at 1,250 °C. For each synthesis, 10 to 40 crystals, including those in *SI Appendix*, Fig. S1, were individually measured. The measured 1/χ were fitted separately to extract the Curie–Weiss temperature, to generate the plotted data in Fig. 2 as the statistical average and distribution of *T*_CW_ in these crystals.

### Neutron Magnetic Diffuse Scattering.

Single-crystal neutron magnetic diffuse scattering was conducted at the time-of-flight beamline BL-9 (CORELLI) of the Spallation Neutron Source, Oak Ridge National Laboratory (37). Two sets of stoichiometric crystals of different synthesis conditions were measured. The first set was a mosaic assembly of 30 crystals, coaligned to ∼6° full width at half maximum (FWHM), with a total mass of 390.0 mg, all grown with *T*_g_ = 1,250 °C and *r* = 2.9. The mosaic assembly was constructed within a volume of 8 × 8 × 8 mm^3^ (*SI Appendix*, Fig. S1*E*), glued by fluoropolymer (CYTOP CTX-809A) on both sides of an aluminum strip (0.3 mm thick). The second set is an individual single crystal with a mass of 7.4 mg, grown with *T*_g_ = 1,000 °C and *r* = 1.75 (*SI Appendix*, Fig. S1*C*), and wrapped in aluminum foil for the measurement.

Each set of crystals was mounted in a close-cycle cryostat with 6 K base temperature. The magnetic diffuse-scattering data were collected by taking 240 exposures of each set of crystals, which was rotated together with the cryostat about the vertical axis over a full 2π range with evenly spaced 1.5° steps. Collected neutron-scattering events in the 240 exposures were used to construct a three-dimensional dataset in the coordinates of reciprocal space, using Python scripts locally developed at CORELLI, together with the Mantid program for visualization (37).

Neutron scattering was measured at three temperatures for each sample, below and immediately above the antiferromagnetic transition temperature *T*_N_ and at high temperature where the spin correlation was no longer observed. For the crystals grown with *T*_g_ = 1,250 °C, magnetic diffuse scattering was measured at 6 K (with a total proton charge of 96 C), 15 K (a total proton charge of 67.2 C), and 100 K (a total proton charge of 57.6 C). For the crystal grown with *T*_g_ = 1,000 °C, magnetic diffuse scattering was measured at 6 K (a total proton charge of 76.8 C), 11 K (a total proton charge of 107.8 C), and 25 K (a total proton charge of 45.6 C). Representative neutron-scattering data before background subtraction are shown in *SI Appendix*, Figs. S2 and S3 for both samples and all temperatures.

To compare magnetic diffuse-scattering patterns of these two sets of crystals (Fig. 3 *A*–*D*), we scaled their relative intensities by the ratio of reciprocal-space integrated intensities of a few reflection families. Two reflection families <1, 1, 1> and <2, 2, 0> are selected due to minimal influence of the extinction effect (Fig. 5) and the fact that their transferred momenta are below that of the lowest aluminum reflection, so contamination of the Al powder ring is avoided during integration. All available reflections, eight of the <1, 1, 1> family and 10 or 12 of the <2, 2, 0> family, are individually integrated to provide an average of each family. For these two families, intensity ratios between the two sample sets are consistent within 10% of each other. The average ratio of 28.2 is used as the scaling factor between the two sets of data and is reasonably consistent with the mass ratio of 52.7 between the two samples sets. The fractional discrepancy could be due to the absorption effect of the larger mosaic assembly (*SI Appendix*, Fig. S1*E* vs. *SI Appendix*, Fig. S1*C*). After the renormalization of scattering intensities at each temperature, Fig. 3 has the high-temperature lattice and background scattering subtracted to isolate the magnetic diffuse-scattering signal.

For a comparison of lattice and magnetic correlation lengths in Fig. 3 *E* and *F*, radial (longitudinal) scans were generated for both the (1, 1, 1) lattice reflection and the (1/2, 1, 0) magnetic reflection. The (1, 1, 1) lattice reflection is chosen to provide a transferred momentum closest to that of the magnetic reflection. For each radial scan, scattering intensities were integrated over two transverse directions. The ranges of integration along the transverse (1, −1, 0) and (1, 1, −2) directions of the (1, 1, 1) lattice reflection are 0.43 × 0.46 Å^−2^ and 0.22 × 0.24 Å^−2^, for samples in *SI Appendix*, Fig. S1 *E* and *C* respectively. The ranges of integration along the transverse (−2, 1, 0) and (0, 0, 1) directions of the (1/2, 1, 0) magnetic reflection are 0.25 × 0.26 Å^−2^ and 0.17 × 0.19 Å^−2^ for samples in *SI Appendix*, Fig. S1 *E* and *C* respectively. The difference in ranges takes into consideration the mosaic assembly (*SI Appendix*, Fig. S1*E*) versus the single crystal (*SI Appendix*, Fig. S1*C*).

### Heat Capacity.

For each growth condition of (*T*_g_, *r*), heat capacity *C*_p_(*T*) of at least two pieces of single crystals was individually measured at zero field, using the heat capacity measurement option (D650) of the physical property measurement system (DynaCool; Quantum Design Inc.). The measurements used the pulse-relaxation scheme, with the size of temperature rise controlled to 0.02*T* at each temperature *T*. Each *C*_p_(*T*) curve was measured at least three times at each temperature for both consistency and statistical averaging. For the calculation of the magnetic entropy, the phonon contribution was removed from the total heat capacity *C*_p_(*T*) in the range of 0 to 50 K, using a *T*^3^ functional form at low temperature, transitioning to a linear form at higher temperature up to 90 K.

### Neutron Single-Crystal Diffraction.

Single-crystal neutron diffraction was measured using a Laue diffractometer at the time-of-flight beamline BL-12 (TOPAZ) of the Spallation Neutron Source, Oak Ridge National Laboratory (33, 34). The beamline utilizes a broad continuous spectrum of incident neutron wavelengths from 0.6 to 3.5 Å, bounded from below by the focusing capability of the neutron guide and from above by the pulsing period of the spallation source and the distance between the beamline and the source. All three stoichiometric crystals (*SI Appendix*, Fig. S1 *B*–*D*) were measured at 100 K, cooled by a liquid nitrogen jet, to minimize the background scattering and reduce the Debye–Waller effect. Each crystal was measured at many different angular positions to provide a nearly complete coverage of reflections for all *d* spacings larger than 0.40 Å. Reflections with *d* < 0.40 Å were not considered in our data analysis. For each crystal in *SI Appendix*, Fig. S1 *B*–*D*, there were 8,176, 13,623, and 13,569 observed reflection events with d≥  0.40 Å, measured over a total flux of 63, 94.3, and 135.9 C of proton charge, respectively. These reflections confirm the cubic symmetry of the UB orientation matrix and can be categorized into 297 unique families under the space group $F\bar{4}3m.$ The number of measured reflections over these 297 families provides a large degree of redundancy over different neutron wavelengths, clearly revealing the extinction effect (Fig. 5*B*). The collected neutron-scattering events were reduced to a list of intensities, each proportional (by an overall coefficient α) to the structure factor $F_{\mathrm{hkl}}^{2}$ of reflection (*h*, *k*, *l*), using Python scripts developed locally at TOPAZ.

In the presence of a strong extinction effect (Fig. 5*B*), we found the calculation for secondary extinction with Gaussian mosaic distribution in type II crystals, in a modified version of equation 37 of ref. 38, best suited our diffraction data:

[1] $$\alpha F_{\mathrm{hkl}}^{2}(\lambda )=m_{1}/{\left(1+2.12m_{1}m_{2}\lambda^{2}+\frac{A\left(\theta \right)m_{1}^{2}m_{2}^{2}\lambda^{4}}{1+B\left(\theta \right)m_{1}m_{2}\lambda^{2}}\right)}^{1/2}.$$

Here, $A\left(\theta \right)=0.58+0.48\cos\;(2\theta )+0.24{\cos\;}^{2}(2\theta )$ and B(θ)=0.02−0.025 cos(2θ) are from equation 43 of ref. 38. The parameter $m_{1}=\alpha F_{\mathrm{hkl}}^{2}(0)$ is a product of the extinction-corrected structure factor $F_{\mathrm{hkl}}^{2}(0)$ and the overall scaling factor α, while the parameter $m_{2}/\alpha$ is indicative of the extinction parameter.

A high-fidelity extraction of $m_{2}$ has posed challenges to our data analysis. As exemplified in Fig. 5*B*, each of the 297 unique reflection families was fitted with Eq. **1** to extract $m_{2}.$ However, for strong reflections, Eq. **1** is not applicable (38), which amounts to about 21 to 26% of the reflection families. In addition, many reflections of small *d* spacing were measured over a very small range of neutron wavelength, which makes it difficult to determine the free-floating value of $m_{2}.$ $m_{2}$ can also be strongly influenced by a low number of redundancies and the counting statistics of weak reflections. Here, we extracted an average $m_{2}$ based on 27 to 31 families (∼10% of total 297 families) with *d* spacing greater than 0.7 Å, all with intermediate reflection strength and measured over a large range of neutron wavelength from 0.6 to over 1.4 Å. All these individual fit values of $m_{2}$ are consistent within 3σ of their respective uncertainties. The fact that $m_{2}$ is a constant for these 27 to 31 reflections that span over a large range of *d* spacing from 0.74 to 4.87 Å justifies the presumption that our crystals are of type II (38). Except for those for which Eq. **1** does not apply, the average $m_{2}$ is applied to 74 to 79% of all reflection families to extract $\alpha F_{\mathrm{hkl}}^{2}(0).$ These extinction-corrected intensities $\alpha F_{\mathrm{hkl}}^{2}(0)$ are further refined with space group $F\bar{4}3m,$ using the Shelxl program, to extract the three lattice parameters of the *B*-site breathing displacement and two oxygen positions along the (1, 1, 1) direction (*SI Appendix*, Table S1).

## Supplementary Material

Supplementary File

## Data Availability

All study data are included in the article and/or *SI Appendix*.
