# Supplementary material for: Controlling inversion disorder in a stoichiometric spinel magnet
Source: Proc Natl Acad Sci U S A. 2022 Oct 18;119(43):e2208748119. doi: 10.1073/pnas.2208748119 (PMC9618041; doi:10.1073/pnas.2208748119)
Supplement: Supplementary File [file pnas.2208748119.sapp.pdf]

PNAS

Supporting Information for

Controlling inversion disorder in a stoichiometric spinel magnet

Margarita G. Dronova[1], Feng Ye[2], Scott E. Cooper[1], Anjana Krishnadas[1], Christina M. Hoffmann[2], Yuita Fujisawa[1], Yoshinori Okada[1], Daniel I. Khomskii[3], Yejun Feng[1,*]

[1]Okinawa Institute of Science and Technology Graduate University, Onna, Okinawa 904-0495, Japan
[2]Neutron Scattering Division, Oak Ridge National Laboratory, Oak Ridge, Tennessee 37831, USA
[3]II. Physikalisches Institut, Universität zu Köln, D-50937 Köln, Germany

* Corresponding author: Yejun Feng.

Email: yejun@oist.jp

This PDF file includes:

Figures S1 to S4

Table S1

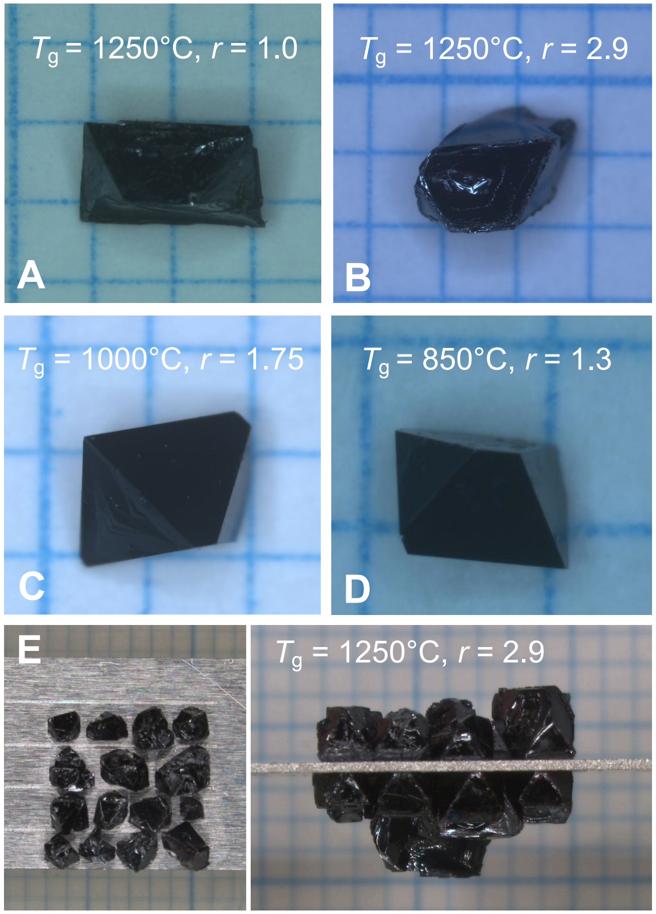

Fig. S1. Single crystal samples. (A-D) Images of single crystals produced under various growth conditions and used in studies of magnetic susceptibility (Fig. 2), heat capacity (Fig. 4), and neutron diffraction refinement at TOPAZ (Fig. 5). The mm-sized grid underneath demonstrates crystals' size. (E) A mosaic crystal assembly for neutron magnetic diffuse scattering study at CORELLI (*Materials and Methods*, Fig. 3), viewed from front (left panel) and side (right panel). The crystal in panel C was also studied for magnetic diffuse scattering at CORELLI (Fig. 3).

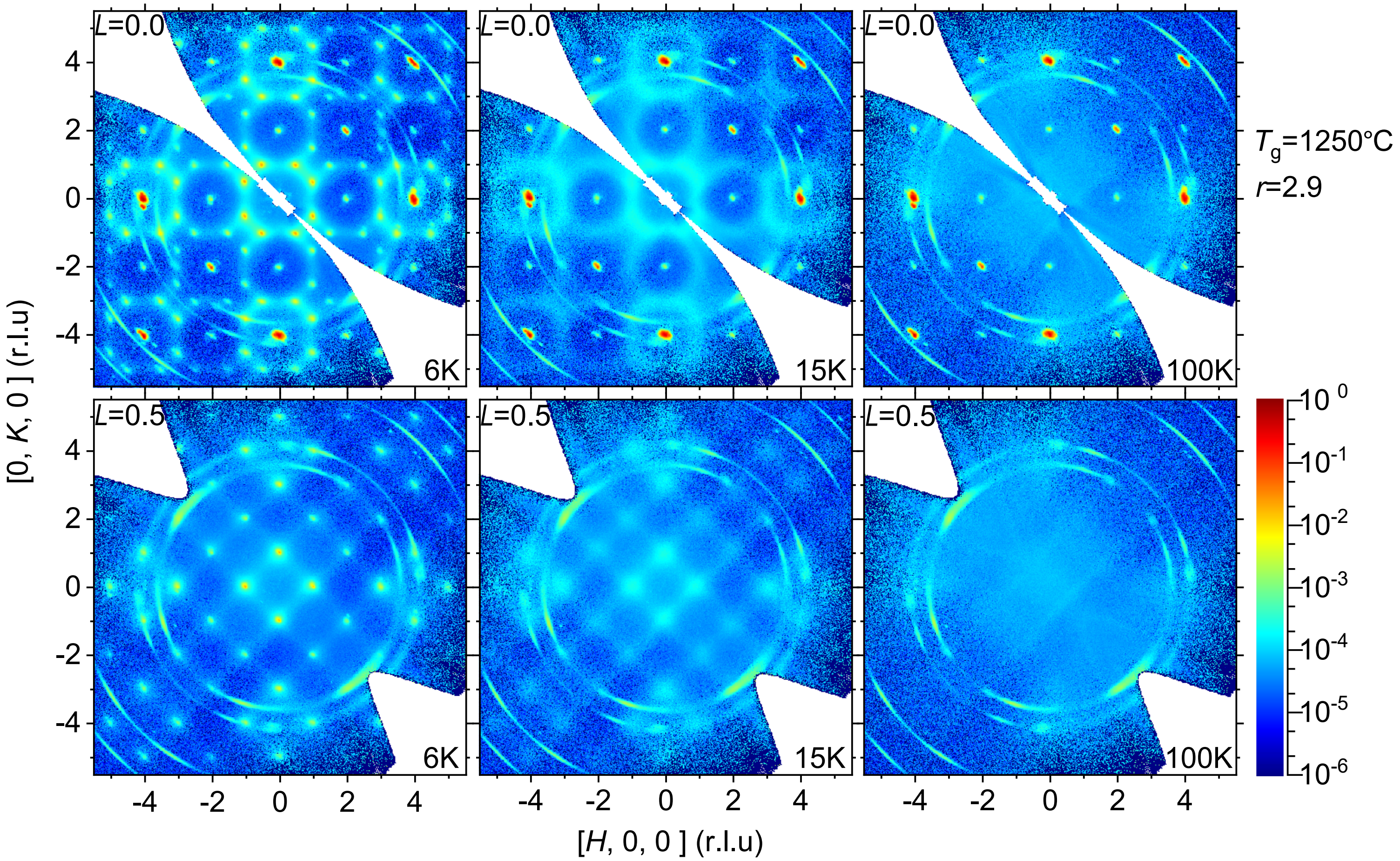

Fig. S2. Neutron magnetic diffuse scattering of $ZnFe_2O_4$ with a high level of inversion disorder. Unsymmetrized scattering intensities that are used to construct Fig. 3*A* and *B*, presented as *H-K* plane slices at *L*=0.0 and 0.5. All intensities have been integrated over a thickness of 0.05 r.l.u. along *L*. The data were measured on a set of crystals grown from T_g =1250 °C (*Materials and Methods*, Fig. S1*E*). Unlike Fig. 3, the data are plotted without subtracting the measurement at high temperature 100 K to reduce the background and the lattice contribution.

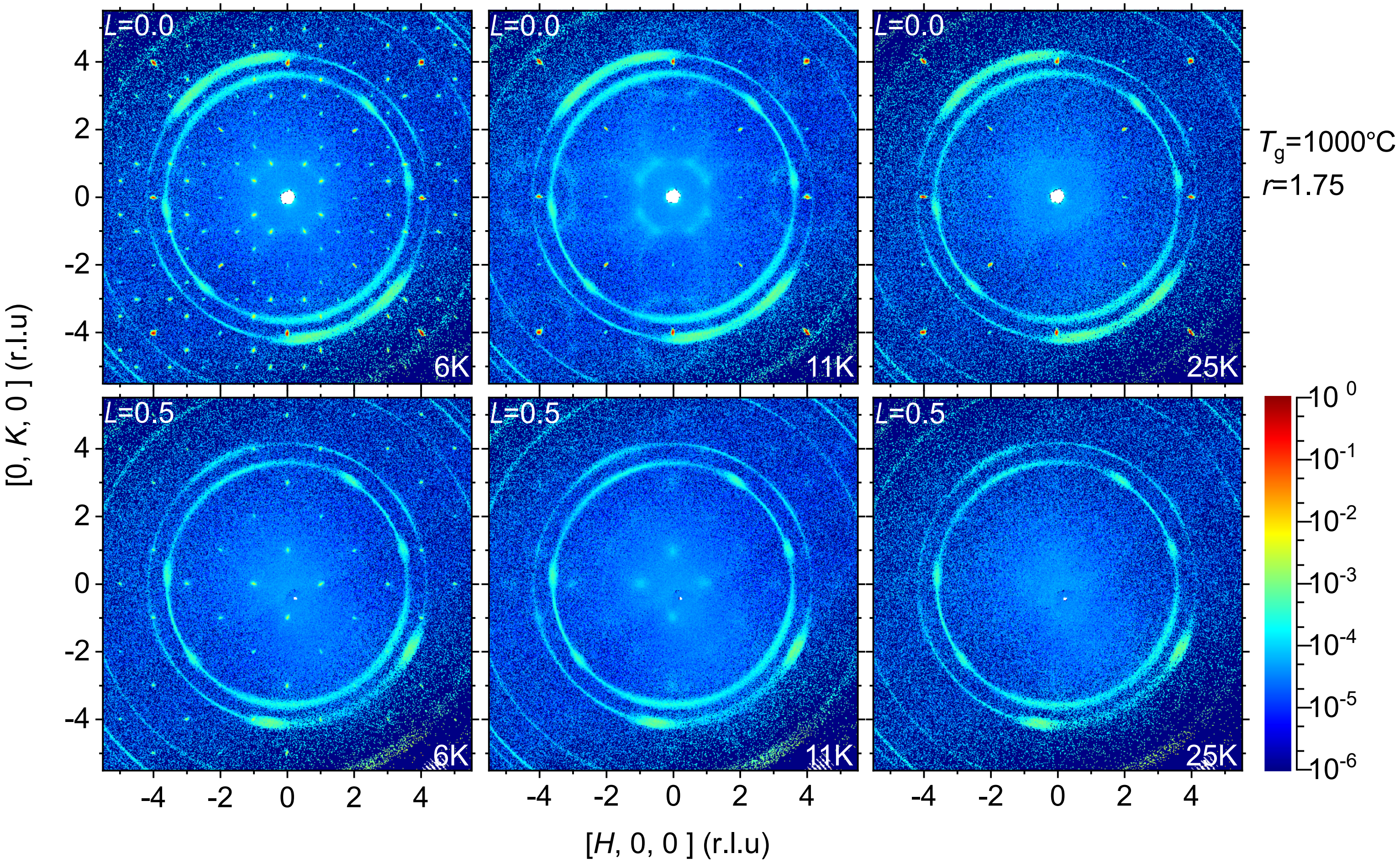

Fig. S3. Neutron magnetic diffuse scattering of $ZnFe_2O_4$ with a low level of inversion disorder. Unsymmetrized scattering intensities that are used to construct Fig. 3 *C* and *D*, presented as *H-K* plane slices at *L*=0.0 and 0.5. All intensities have been integrated over a thickness of 0.05 r.l.u. along *L*. The data were measured on an individual piece of crystal grown from T_g =1000 °C (*Materials and Methods*, Fig. S1*C*). Unlike Fig. 3, the data are plotted without subtracting the measurement at high temperature 25 K to reduce the background and the lattice contribution.

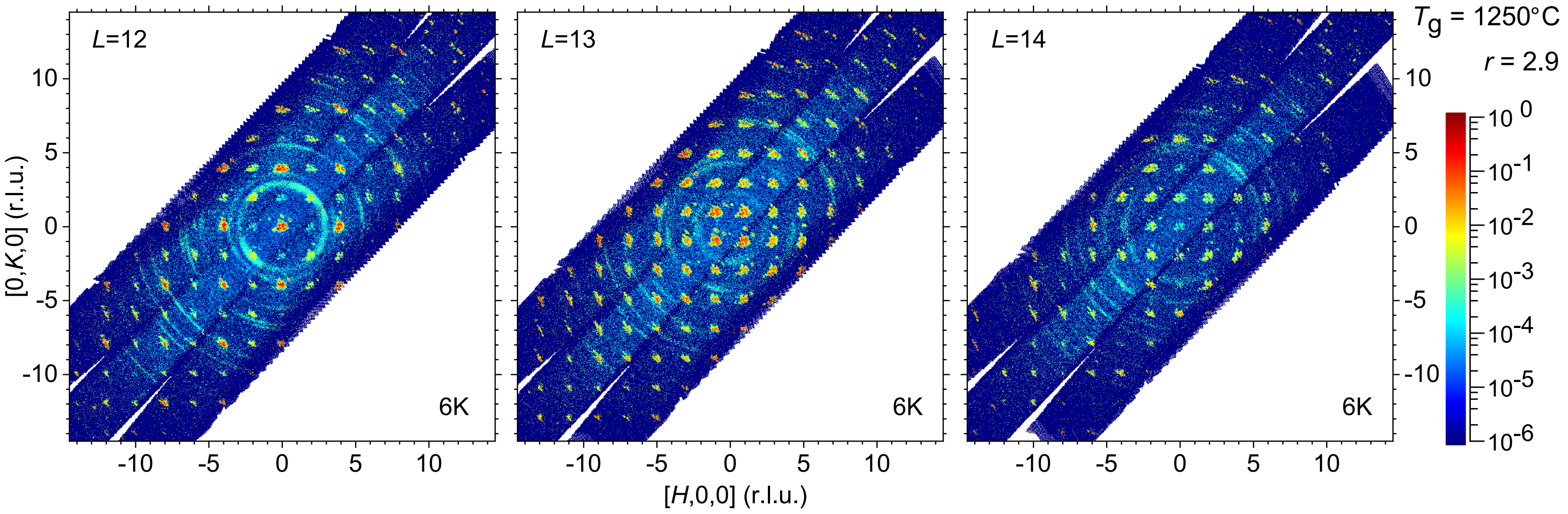

Fig. S4. Neutron diffuse scattering of $ZnFe_2O_4$ crystals with a high level of inversion disorder. Unsymmetrized scattering patterns presented as *H*-*K* plane slices at *L* = 12, 13, and 14 r.l.u. respectively. All intensities have been integrated over a thickness of 0.05 r.l.u. along *L*. The data were measured at 6 K on a set of crystals grown from T_g =1250 °C (*Materials and Methods*, Fig. S1 *E*).

Table S1. Structure parameters of $ZnFe_2O_4$ refined from neutron single crystal diffraction at TOPAZ, using a subset of data after the extinction effect is corrected (*Materials and Methods*). The refinement is modeled for the normal spinel structure of the $F\bar{4}3m$ space group (#216), without parametrizing the inversion disorder. Site occupancies are assumed unity for all three stoichiometric specimens. All data was collected at 100 K.

	Atom	Site	x	y	z	U_{iso}
T_g = 850 °C, r = 1.3	Zn	4a	0	0	0	0.00048(43)
	Zn	4c	0.25	0.25	0.25	0.00207(49)
a = 8.4336(1) Å	Fe	16e	0.62357(9)	0.62357(9)	0.62357(9)	0.00029(21)
	O1	16e	0.38633(21)	0.38633(21)	0.38633(21)	0.00182(26)
R = 0.0807	O2	16e	0.86529(18)	0.86529(18)	0.86529(18)	0.00141(25)
T_g = 1000 °C, r = 1.75	Zn	4a	0	0	0	0.00088(40)
	Zn	4c	0.25	0.25	0.25	0.00258(46)
a = 8.4333(1) Å	Fe	16e	0.62353(8)	0.62353(8)	0.62353(8)	0.00083(20)
	O1	16e	0.38640(19)	0.38640(19)	0.38640(19)	0.00234(25)
R = 0.0664	O2	16e	0.86547(16)	0.86547(16)	0.86547(16)	0.00192(24)
T_g = 1250 °C, r = 2.9	Zn	4a	0	0	0	0.00115(42)
	Zn	4c	0.25	0.25	0.25	0.00241(48)
a = 8.4328(1) Å	Fe	16e	0.62362(9)	0.62362(9)	0.62362(9)	0.00100(23)
	O1	16e	0.38638(20)	0.38638(20)	0.38638(20)	0.00254(28)
R = 0.0765	O2	16e	0.86545(17)	0.86545(17)	0.86545(17)	0.00202(26)
